# Minimal change disease following the Moderna COVID-19 vaccine: first case report

**DOI:** 10.1186/s12882-021-02583-9

**Published:** 2021-11-11

**Authors:** Shaefiq Thappy, Sherin R. Thalappil, Shahem Abbarh, Abdulrahman Al-Mashdali, Mohammed Akhtar, Mohamad M. Alkadi

**Affiliations:** 1 Division of Nephrology, Department of Medicine, Hamad Medical Corporation, Doha, Qatar; 2 Division of Allergy and Immunology, Department of Medicine, Hamad Medical Corporation, Doha, Qatar; 3 Department of Medicine, Hamad Medical Corporation, Doha, Qatar; 4 Department of Pathology, Hamad Medical Corporation, Doha, Qatar

**Keywords:** Nephrotic syndrome, Immunization., Minimal change disease, Case report

## Abstract

**Background:**

There have been cases of minimal change disease (MCD) reported following previous vaccines. During the COVID-19 era, only 3 cases of new-onset MCD and a case of MCD relapse were reported following the Pfizer-BioNTech COVID-19 vaccine. We herein report the first case of MCD after receiving the Moderna COVID-19 vaccine.

**Case presentation:**

A 43-year-old man presented to hospital 3 weeks after receiving the first dose of the Moderna vaccine, with both bilateral lower extremities and scrotal edema. He initially developed a sudden-onset bilateral lower extremities swelling on day 7 post-vaccine. He, then, developed dyspnea and scrotal swelling over a time span of 2 weeks. On physical examination, his blood pressure was 150/92 mmHg. There was a decreased air entry at lung bases, bilateral lower extremities and scrotal edema. Labs revealed hypoalbuminemia, hyperlipidemia and 15 g of proteinuria. His immunologic and serologic work up was negative. Renal biopsy showed concomitant MCD and IgA nephropathy. Patient was treated with oral steroids and had a good response; his edema resolved, serum albumin improved, and proteinuria decreased to 1 g within 2 weeks of treatment.

**Conclusions:**

To the best of our knowledge, MCD has not been previously reported after receiving the Moderna COVID-19 vaccine. It remains unclear whether the COVID-19 mRNA vaccines are associated with the development of MCD, or it coincided with the mass vaccination. Further studies are needed to determine the incidence of MCD post COVID-19 vaccines and the underlying pathophysiology of glomerular injury post vaccination.

## Background

Since its emergence in December 2019, the coronavirus disease (COVID-19) has spread all over the world resulting in major crises in health systems and the global economy [1, 2]. According to World Health Organization, there have been around 175 million cases of COVID-19 globally, causing more than 3.7 million deaths since the start of this pandemic [3]. In an effort to control the spread of COVID-19 and to reduce the severity of the disease as well as the risk of death, several vaccines have been developed. Despite all the undoubted advantages and benefits of the newly developed COVID-19 vaccines, a few serious adverse incidents are emerging with the increasing number of people receiving the vaccines.

Renal complications have been observed following several previous vaccines [4]. For instance, minimal change disease (MCD) has been reported following vaccines against hepatitis, pneumococcus, influenza and tetanus-diphtheria-poliomyelitis [5–8]. In the COVID-19 era, 3 cases of new-onset MCD and one case of MCD relapse have been published in the literature following the Pfizer-BioNTech COVID-19 vaccine [9–12]. We herein report the first case of MCD after receiving the first dose of the Moderna COVID-19 vaccine.

## Case presentation

A 43-year-old Ethiopian man, with no significant past medical history, presented to our hospital, 3 weeks after receiving the first dose of Moderna COVID-19 vaccine, with both bilateral lower extremities and scrotal edema. He initially developed a sudden-onset bilateral lower limb swelling on day 7 post-vaccine. Following on from that, he developed dyspnea on exertion and scrotal swelling over a time span of 2 weeks. He denied fever, chills, nasal congestion, sore throat, cough, chest pain, vomiting, diarrhea, skin rash, joint pain, change in the color or amount of urine, hematuria or dysuria. The patient did not report any recent viral infection, and he did not travel or come in contact with any sick people. Furthermore, he denied taking any herbals or over the counter medications such as non-steroidal anti-inflammatory drugs. The patient also denied having a similar episode in the past or having a family history of kidney diseases. He is married and works in an office. He occasionally drinks alcohol, but he does not smoke cigarettes.

Upon presentation, his temperature was 36.8 degree Celsius, blood pressure was 150/92 mmHg, heart rate was 87 beats per minute, respiratory rate was 18 breaths per minute and oxygen saturation was 98% on room air. On physical examination, he had a decreased air entry at both lung bases, and massive bilateral lower limb pitting edema extending to above the knees and scrotal swelling. The rest of the exam was unremarkable with no palpable lymph nodes. The initial laboratory investigations (Table 1) revealed serum creatinine of 80 μmol/L, blood urea nitrogen of 3.9 mmol/L and albumin of 8 g/L. His urinalysis showed 3+ protein and 2+ blood. He only had 4 red blood cells per high power field on urine microscopy, while his 24-h urine protein was 15 g. His immunologic workup, including complements (C3, C4), antinuclear and anti-phospholipase A2 antibodies, was normal. Additionally, HIV, hepatitis B, C and nasopharyngeal COVID-19 polymerase chain reactions were all negative. Chest x-ray revealed prominent vascular markings and mild bilateral pleural effusion, while abdominal ultrasound showed normal-sized kidneys with normal echogenicity and corticomedullary differentiation. The patient did not have previous lab results or imaging prior to this hospitalization.

**Table 1:** Laboratory tests on admission and after steroids therapy

Laboratory Test	Admission	14 days after steroids
Blood urea nitrogen, mmol/L	3.9	5
Creatinine, μmol/L	80	61
Albumin, g/L	8	25
Total Cholesterol, mmol/L	10.8	–
Triglycerides, mmol/L	2.3	–
HDL, mmol/L	1.2	–
LDL, mmol/L	8.6	–
24-h urine protein, gram	15	1

**Fig. 1:**
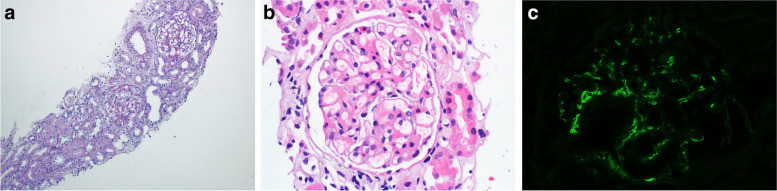
Kidney biopsy (**A**) PAS stain (200 x) showing essentially normal renal parenchyma, one artery appears unremarkable. The glomerulus reveals mild mesangial matrix expansion (**B**) H&E Stain (300 x) showing mild mesangial expansion and patent capillary lumens (**C**) Immunofluorescence stain revealing 2 + Mesangial deposition of IgA

The patient was started on furosemide and amlodipine and later had a kidney biopsy. There were 14 glomeruli, on light microscopy, with mild mesangial proliferation and expansion; however, none of the glomeruli had endocapillary proliferation, segmental glomerulosclerosis or crescents (Fig. 1A, B). Also, there was no tubular atrophy or interstitial fibrosis. Direct immunofluorescence study revealed 2+ mesangial deposition of immunoglobulin A (IgA), trace immunoglobulin G (IgG) and 1+ C3 (Fig. 1C). However, electron microscopy was not performed due to unavailability. The patient’s nephrotic syndrome was attributed to MCD and he was treated with oral prednisolone 80 mg daily. When seen in the clinic, after 2 weeks of steroids, he had no lower extremities or scrotal edema. In addition, his serum albumin increased to 25 g/L and his 24-h urine protein was down to 1 g. The patient tolerated treatment well without side effects. He was advised to continue prednisolone 80 mg daily to complete a total of 4 weeks and then his prednisolone dosage will be tapered down over the following 4 months under close monitoring in clinic.

## Discussion and conclusions

MCD accounts for up to 25% of nephrotic syndrome cases in adults. However, only a few cases of MCD post vaccination have been reported with symptoms starting 4 days to 16 weeks after vaccination [8]. Since the start of COVID-19 pandemic, 3 cases of new-onset MCD have been reported in adult men after receiving the Pfizer-BioNTech COVID-19 vaccine [9–11]. These cases were similar to our case in that the symptoms developed 3 to 7 days after receiving the first dose of a COVID-19 messenger ribonucleic acid (mRNA) vaccine and they had a good response to steroids. However, unlike these cases, our patient did not present with acute kidney injury and his kidney biopsy showed concomitant IgA nephropathy.

Although the pathogenesis of MCD is not fully understood, several studies suggest that T cell dysfunction might be the main underlying immunological mechanism. The dysfunction of T cells may result in production of a glomerular permeability factor that alters glomerular permeability and causes diffuse foot process effacement and marked proteinuria. Moreover, the involvement of both immature CD34+ T cells and effector T cells (helper, Cytotoxic and regulatory T cells) has also been established [13–16]. Normally, after vaccination, the vaccine’s antigen is taken up by dendritic cells and then presented to T cell receptors on naïve T cells. This results in the activation of antigen-specific effector T cells that peak between 7 and 14 days after vaccination [17]. Our patient developed symptoms 7 days after receiving the first dose of COVID-19 vaccine, which may correspond to the time period when antigen-specific T cells peak after vaccination.

Nephrotic syndrome in IgA nephropathy is rare and is usually evident in patients with endocapillary proliferation, segmental sclerosis and/or crescents. Moreover, it is often resistant to steroid therapy [18]. The nephrotic syndrome in our patient was attributed to MCD rather than IgA nephropathy as he only had mild mesangial proliferation on kidney biopsy, and his nephrotic syndrome responded well to corticosteroids. It is worthy to note that the one limitation of our case report is that there was no electron microscopy available to show diffuse effacement of podocytes’ foot processes.

To the best of our knowledge, this is the first reported case of MCD following the Moderna COVID-19 vaccine. However, it remains unclear whether COVID-19 mRNA vaccines are associated with the development of MCD, or it coincided with the mass vaccination. Also, it is not clear whether the second dose of Moderna COVID-19 vaccine should be given to patients who develop MCD post vaccine. Further studies are needed to determine the incidence of MCD post COVID-19 vaccines and attempt to explain the pathophysiology of glomerular injury post vaccination.

## Data Availability

Data sharing is not applicable to this article as no datasets were generated or analysed during the current study.

## Abbreviations

COVID-19: Coronavirus disease 2019
C3: Complement 3
C4: Complement 4
g/L: Gram per liter
HIV: Human immunodeficiency virus
IgA: Immunoglobulin A
IgG: Immunoglobulin G
MCD: Minimal change disease
mm Hg: Millimeter of mercury
μmol/l: Micromole per liter
mmol/L: Millimole per liter
mRNA: Messenger ribonucleic acid
